# Pulmonary hydatid cyst in a pregnant patient causing acute respiratory failure

**DOI:** 10.4103/1817-1737.32234

**Published:** 2007

**Authors:** Mohammed H. Hijazi, Mariam A. Al-Ansari

**Affiliations:** *Section of Critical Care Medicine, Department of Medicine, King Faisal Specialist Hospital and Research Centre, Riyadh, Saudi Arabia*

**Keywords:** Critical illness, pregnancy, pulmonary hydatid, tuberculosis

## Abstract

A 21-year-old primigravida, at 32 weeks of gestation, presented with acute onset of respiratory failure and circulatory shock. Chest imaging showed findings suggestive of ruptured hydatid cyst, which was confirmed by histology post-thoracotomy. Tissue cultures from the removed cyst grew Mycobacterium tuberculosis also. She was successfully managed in the intensive care unit and was then discharged home on antituberculosis medications in addition to albendazole after prolonged hospitalization and a need for chest tube for bronchopleural fistula. Acute respiratory failure and anaphylactic shock secondary to ruptured pulmonary hydatid cyst and superimposed pulmonary tuberculosis in a pregnant lady should be considered in patients living in endemic areas.

Critical illness requiring admission to an intensive care unit (ICU) during pregnancy is relatively uncommon.[1] Respiratory failure and homodynamic instability are responsible for up to 80% of the obstetric admissions to the ICU. On the other hand, hydatid disease in Saudi Arabia affects females more than males.[2] Acute presentation because of ruptured cyst is uncommon. While unreported before, superimposed tuberculosis infection might play a role in precipitating rupture. We report an unusual case of a primigravida with ruptured pulmonary hydatid disease and superimposed tuberculosis presenting with acute respiratory failure and shock requiring ICU admission.

## Case Report

A 21-year-old primigravida, at 32 weeks of gestation, was transferred to the ICU at King Faisal Specialist Hospital and Research Centre as a case of respiratory failure secondary to pneumonia. She had history of sudden onset shortness of breath, right-sided pleuritic chest pain and wheezes for 1 day preceded by cough, yellow sputum and shortness of breath of 1 week duration. She denied any other history like fever or orthopnea. She is a nonsmoker. There was no recent history of animal contact. Her physical examination revealed severe respiratory distress with a respiratory rate of 35/min, tachycardia of 120/min, fever of 38.0°C, blood pressure of 100/60 mmHg, decreased breach sounds at the right base and wheezes all over the chest. Oxygen saturation was 90% on nonre-breather facemask (NRFM) at 15 L/min. Rest of the examination was unremarkable apart from a gravid uterus with viable fetus.

**Figure 1:**
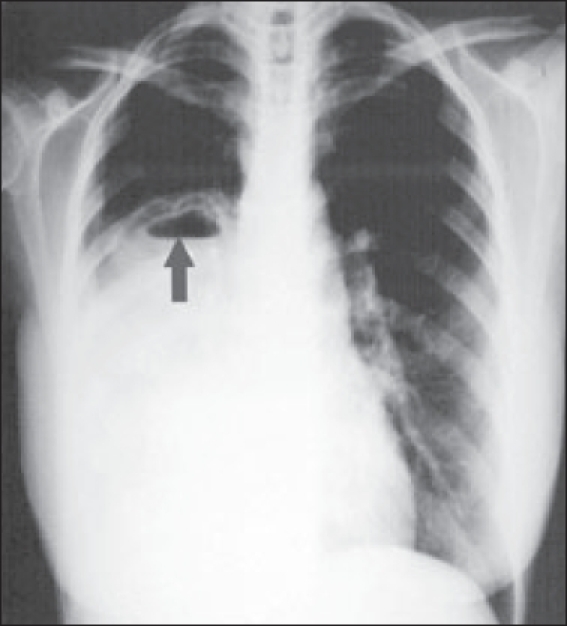
Chest radiograph at initial presentation showing uniform opacification of right lower zone, which mimicked pleural effusion and obscured the underlying cyst with air fluid level (gray arrow)

Arterial blood gases on NRFM at 15 L/min showed pH 7.24, PaCO2 6.2 kPa, PaO2 14.3 kPa, HCO3 19 mmol/L and anion gap of 12. Lactic acid was 1.7 mmol/L. Renal and hepatic profiles were normal. Chest X-ray taken on presentation to the referring hospital revealed right pleural effusion with air fluid level [Figure 1]. Thoracentesis done in the referring hospital showed high eosinophil count. Based on these findings, the working diagnoses were complicated pneumonia (with abscess formation), ruptured hydatid cyst and pulmonary embolism with cavity formation. The patient was intubated and nursed on her left lateral position. Mechanical ventilation was set to maintain normal pH and PaCO_2_, plateau pressure of 30 cm H_2_ O or less; and to avoid auto PEEP.

**Figure 2:**
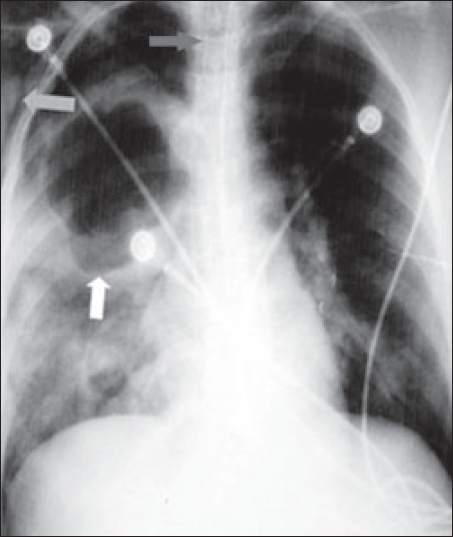
Post intubation chest X-ray showing endotracheal tube (dark gray arrow), ruptured cyst (white arrow), and surgical emphysema (light gray arrow)

Post-intubation chest X-ray [Figure 2] revealed a right lung cavity, disappearance of the air fluid level, subcutaneous emphysema and a pneumothorax on the right side necessitating chest tube insertion.

She soon developed hypotension requiring fluid resuscitation with no evidence of tension pneumothorax. Despite keeping the patient on the left lateral position and maintaining adequate central venous pressure (14 mmHg), blood pressure continued to drop, requiring vasopressors to keep the MAP above 65 mmHg. After completing full septic work-up, broad-spectrum antibiotics and intravenous hydrocortisone were started to treat any element of infection and anaphylaxis that might be associated with ruptured hydatid cyst. After stabilization, the patient was taken for CT scan of the chest, which revealed three cysts involving the right lung with typical ‘water-lily’ sign in one of them, as well as two cystic lesions involving the liver. Serology for hydatid cyst was highly positive (1:6348). A decision between the intensivist, thoracic surgeon, obstetrician and a neonatologist was made to proceed with caesarian section followed by right thoracotomy to save both the mother and the baby. Intraoperatively, three hydatid cysts were resected from the right upper, lower and middle lobes.

Postoperatively, the patient improved markedly, was off pressors and mechanical ventilation in 2 days and was discharged to the floor after 5 days. Histopathology showed multiple endocyts with viable germinal epithelium and scoleces, confirming the diagnosis of hydatid disease. She continued to have the right chest tube in place (removed 3 weeks later) because of large air leak secondary to bronchopleural fistula. She was eventually discharged home on albendazole. Two weeks later, tissue culture from the ruptured hydatid cyst grew Mycobacterium tuberculosis sensitive to first-line therapy. She was treated with four antituberculosis medications, and the baby was referred to the pediatric infectious disease specialist. A year later, both patient and the infant were alive and well. Follow-up chest X-ray was normal.

## Discussion

This is a rare case of ruptured pulmonary hydatid cyst with superimposed tuberculosis presenting with acute respiratory failure and anaphylactic shock during pregnancy. To the best of our knowledge, this is the first report of such a case. The diagnosis was not entertained initially and the patient was treated as complicated community-acquired pneumonia by the referring hospital. The initial chest X-ray showed a right-sided cavity with air fluid level that disappeared on the subsequent X-ray. This rapid change was consistent with a cavity that ruptured and emptied its contents. The diagnosis of ruptured hydatid cyst was entertained and confirmed later by the classic finding on the CT scan. The acute hypoxemic respiratory failure is possibly secondary to parenchymal injury as well as acute bronchospasm secondary to ruptured cyst, as suggested by the rapid resolution after starting hydrocortisone, bronchodilators and cyst resection. The most likely explanation for the shock is anaphylaxis secondary to the ruptured hydatid cyst. All initial bacterial cultures were negative.

Few weeks later, the tissue culture from the resected cyst was, surprisingly, positive for *Mycobacterium tuberculosis*. It is possible that the decrease in cellular immunity that accompanies pregnancy might be responsible for the progression of both the hydatid disease and the superimposed tuberculosis, which in turn could have played a role in precipitation of rupture.

Hydatid disease in pregnancy is a rare condition, with an incidence of 1/20,000 to 1/30,000 deliveries.[3,4] In late pregnancy, it carries a risk of enlargement with subsequent rupture and anaphylactic shock.[5]

The diagnosis of pulmonary hydatid cyst should be entertained based on radiographic appearance and epidemiological setting.[6] The results of standard serologic assays are positive in only 50-60% of cases with lung hydatid cysts.[7] Treatment of pulmonary hydatid cyst is essentially surgical.[8] Chemotherapy (mebendazole and albendazole) is used as a complement to operative treatment to avoid recurrence.[9]

Critical illness during pregnancy demands a multidisciplinary approach in order to optimize maternal and fetal outcomes. The uteroplacental blood flow, fetus- and pregnancy-associated physiologic changes should be considered when caring for the critically ill pregnant patient.[10] Permissive hypercapnia is not an option às maternal hypercapnia quickly results in fetal respiratory acidosis.[11] Indications for invasive hemodynamic monitoring in obstetric patients are much the same as any other patient.[12] Hypotension management needs particular attention to body position and volume status. Special attention is needed to avoid fetal exposure to toxic medications (like vasopressor/inotrop) and unnecessary radiation.

Ruptured pulmonary hydatid cyst should be considered in patients living in endemic areas with suggestive radiologic findings, especially during pregnancy. The need for a multidisciplinary team approach to optimize maternal and fetal outcomes in a critically ill pregnant patient requiring treatment in an ICU cannot be re-emphasized.
